# Metabolomic and transcriptomic analyses reveal new insights into the role of abscisic acid in modulating mango fruit ripening

**DOI:** 10.1093/hr/uhac102

**Published:** 2022-05-10

**Authors:** Shibo Wu, Di Wu, Juan Song, Yanyu Zhang, Qing Tan, Tianquan Yang, Jingya Yang, Songbiao Wang, Jianchu Xu, Wei Xu, Aizhong Liu

**Affiliations:** Key Laboratory of Economic plants and Biotechnology, Kunming Institute of Botany, Chinese Academy of Sciences, 132 Lanhei Road, Kunming 650201, China; University of Chinese Academy of Sciences, Beijing 100049, China; Key Laboratory of Economic plants and Biotechnology, Kunming Institute of Botany, Chinese Academy of Sciences, 132 Lanhei Road, Kunming 650201, China; University of Chinese Academy of Sciences, Beijing 100049, China; Key Laboratory of Economic plants and Biotechnology, Kunming Institute of Botany, Chinese Academy of Sciences, 132 Lanhei Road, Kunming 650201, China; Key Laboratory of Economic plants and Biotechnology, Kunming Institute of Botany, Chinese Academy of Sciences, 132 Lanhei Road, Kunming 650201, China; University of Chinese Academy of Sciences, Beijing 100049, China; Key Laboratory of Economic plants and Biotechnology, Kunming Institute of Botany, Chinese Academy of Sciences, 132 Lanhei Road, Kunming 650201, China; University of Chinese Academy of Sciences, Beijing 100049, China; Germplasm Bank of Wild Species, Kunming Institute of Botany, Chinese Academy of Sciences, Kunming 650201, China; Key Laboratory of Economic plants and Biotechnology, Kunming Institute of Botany, Chinese Academy of Sciences, 132 Lanhei Road, Kunming 650201, China; University of Chinese Academy of Sciences, Beijing 100049, China; Key Laboratory of Tropical Fruit Biology of Ministry of Agriculture, South Subtropical Crops Research Institute, Chinese Academy of Tropical Agricultural Sciences, Zhanjiang 524091, China; Key Laboratory of Economic plants and Biotechnology, Kunming Institute of Botany, Chinese Academy of Sciences, 132 Lanhei Road, Kunming 650201, China; Key Laboratory of Economic plants and Biotechnology, Kunming Institute of Botany, Chinese Academy of Sciences, 132 Lanhei Road, Kunming 650201, China; Key Laboratory for Forest Resource Conservation and Utilization in the Southwest Mountains of China, Ministry of Education, Southwest Forestry University, Kunming 650224, China

## Abstract

Mango (*Mangifera indica* L.) is a climacteric tropical fruit consumed around the world. Although ethylene and abscisic acid (ABA) have been considered to be stimulators that trigger mango fruit ripening, their regulation mechanisms in modulating mango fruit ripening remain uncertain. In this study, we performed integrative analyses of metabolome and transcriptome data combined with a series of physiological and experimental analyses in the ‘Keitt’ mango, and we characterized changes in accumulation of specific metabolites at different stages during fruit development and ripening, which were strongly correlated with transcriptional changes and embodied physiological changes as well as taste formation. Specifically, we found that ABA, rather than ethylene, was highly associated with mango ripening, and exogenous ABA application promoted mango fruit ripening. Transcriptomic analysis identified diverse ripening-related genes involved in sugar and carotenoid biosynthesis and softening-related metabolic processes. Furthermore, networks of ABA- and ripening-related genes (such as *MiHY5*, *MiGBF4*, *MiABI5*, and *MibZIP9*) were constructed, and the direct regulation by the key ABA-responsive transcription factor MiHY5 of ripening-related genes was experimentally confirmed by a range of evidence. Taken together, our results indicate that ABA plays a key role in directly modulating mango fruit ripening through MiHY5, suggesting the need to reconsider how we understand ABA function in modulating climacteric fruit ripening.

## Introduction

Fruit ripening is a highly complicated, well-coordinated and finely regulated biological process usually associated with a series of changes in taste (sweetness and acidity), texture (softening and firmness), and appearance (color) [1–5]. Dissecting the physiological and molecular mechanisms behind the fruit ripening process will not only be significant for understanding biological and evolutionary contexts with regard to seed development and dispersal, but also indispensable for developing fruit safety and meeting fruit supply demands at markets across different seasons by regulating the asynchronous development and ripening processes of different varieties [3, 6–8].

Although fruit species and types are diverse, fruits are, in general, categorized into two types: climacteric (such as tomato) and non-climacteric fruits (such as grape) depending on whether there is a characteristic burst of respiration during fruit development [2, 3, 9–11]. It is commonly believed that climacteric fruit ripening is mainly triggered by elevated ethylene levels during fruit development and ripening, while the ripening of non-climacteric fruits is usually activated by an increase in abscisic acid (ABA) production with fruit development [12]. The involvement of ethylene in fruit ripening has been well documented, and changes in ethylene accumulation with fruit development greatly impact physiological responses, signal transduction, and gene transcription, triggering the processes of fruit ripening [3, 13]. Furthermore, several key transcription factors (TFs) such as ERFs, CNR, and RIN in the ethylene pathway have been identified to be critical for modulating the processes of fruit ripening by changing specific traits, including color, firmness, aroma, taste, and postharvest shelf life [14]. In addition, ABA accumulation has been shown to be critical in mediating non-climacteric fruit ripening in grapes [15] and strawberries [16]. Changes in ABA accumulation directly reshaped the processes behind non-climacteric fruit ripening [16–18]. Usually, ABA regulates the physiological and biochemical changes in fruits by activating TFs such as bZIP, which binds ABA-response elements (ABREs) [19–22].

Increasing evidence has shown that ABA accumulation in climacteric fruits (such as tomato, fig, and peach) participates in the regulation of and affects the processes behind fruit ripening [12, 23, 24]. Specifically, the ABA signal triggers ethylene biosynthesis or interacts with the ethylene signal, clearly affecting the fruit ripening processes in tomato, fig, and banana fruits [25]. It seems that the function of ABA varies in the regulation of fruit ripening in different climacteric fruits. However, the potential physiological and molecular mechanism underlying the participation of ABA in the regulation of fruit ripening largely remains unknown in climacteric fruits. As high-throughput technology has been rapidly developed, integration of multi-omics data has provided a powerful approach to the dissection of the physiological and molecular mechanisms of fruit ripening in diverse fruits [26–31].

Mango (*Mangifera indica* L., Anacardiaceae), a typical tropical perennial fruit, is regarded as ‘The King of Fruits’ for its captivating color, pleasant aroma, delicate taste and diverse nutrient composition, containing vitamins, minerals, and antioxidants [32–35]. Owing to its popularity worldwide, mango has been widely cultivated in tropical and subtropical regions and is now the second most-produced tropical fruit in the world [36]. Previous studies investigating physiological changes in fruit development and ripening have mainly focused on sugar and organic acids, nutrient and flavor compositions, and their antioxidant activities with fruit development and ripening [37]. Since its ripening process is strongly regulated by ethylene, mango is considered a typical climacteric fruit [38]. Interestingly, several studies have noted that increased ABA accumulation with mango fruit ripening [39] and exogenous application of ABA seem to affect the fruit ripening of mango [40]. However, the physiological and molecular mechanisms behind how ABA affects mango fruit ripening remain unclear.

In this study, we performed integrative analyses of physiological investigations, metabolome and transcriptome data, and used experimental confirmation to comprehensively understand the physiological and molecular mechanism of fruit development and ripening in the late-ripening ‘Keitt’ mango. Of note is that we found ABA accumulation was strongly associated with an increase in sweetness and decrease in acidity with the ripening of mango. By a combination of weighted gene co-expression network analysis (WGCNA) method with experimental analysis, it is revealed for the first time that the bZIP-type TF MiHY5, via the ABA pathway, is a key regulator of the fruit ripening of mango. The current study provides a novel insight into understanding the essential function of ABA in climacteric fruit ripening.

## Results

### Observation and characterization of fruit development and ripening

**Figure 1:**
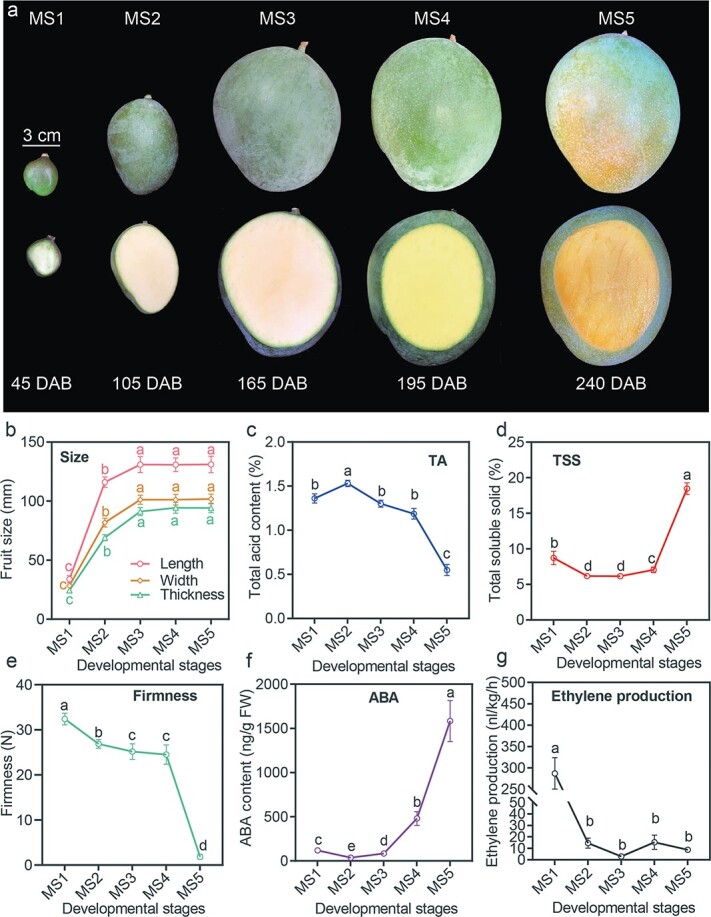
Characterization of ‘Keitt’ fruit growth and development. **a** Phenotypic changes in fruit size and coloration at five developmental stages. Scale bar = 3 cm. **b**–**g** Details of changes in fruit size (length, width, and thickness) (**b**), total acid content (**c**), total soluble solids (**d**), firmness (**e**), ABA content (**f**), and ethylene production (**g**) during ‘Keitt’ fruit development and ripening. Different letters stand for statistically significant differences in different stages calculated through one-way ANOVA (*P* < .05). Error bars stand for the standard deviation of replicates.

We observed the time of flowering and fruit development and ripening, sampling the developing fruit every 15 days after the appearance of the first flower until the fruit fully ripened (Supplementary Data Fig. S1a). Based on changes in fruit weight (Supplementary Data Fig. S1b), we divided fruit development and ripening into five distinct stages: the early fruitlet stage [0–60 days after full bloom (DAB)]; the fast-enlarging stage (60–135 DAB); the early stage of fruit ripening (135–180 DAB, also called the mature green stage); the middle stage of fruit ripening (180–210 DAB); and the late stage of fruit ripening (210–240 DAB). We sampled pulp tissues from developing fruits at 45, 105, 165, 195, and 240 DAB, representing each of the five stages, and recorded them as MS1–MS5 (Fig. 1a), respectively. Furthermore, we recorded fruit size and collected pulp tissues according to Supplementary Data Fig. S1c. We clearly observed that fruit development from MS1 to MS3 was followed by a stable increase in dimensions and weight (illustrated in Fig. 1b and Supplementary Data Fig. S1b), reaching maximum values at MS3; fruit ripening from MS3 to MS5 featured accelerated ripening, and from MS4 to MS5 the fruit was associated with changes in peel color from deep green, present during the MS3 stage, to yellow at the MS5 stage.

We also investigated the physiological changes that occurred in the developing fruit across different stages, including total acid content (TA), total soluble solids (TSS), firmness, ABA content, and ethylene production. A sharp TA decrease in mango pulp was observed from MS4 to MS5 (1.19 to 0.55%) (Fig. 1c), and TSS differed slightly from MS1 to MS4, before substantially increasing (~1.6-fold) from MS4 to MS5, reaching 18.5% at MS5 (ripening stage) (Fig. 1d). Firmness gradually decreased with fruit development and ripening, and sharply reduced from MS4 to MS5 associated with ripening (Fig. 1e). As a typical climacteric fruit, we observed that ABA content gradually increased and reached the maximum at MS5 (1583.5 ng/g fresh weight) (Fig. 1f). In particular, there was a 3.3-fold increase in ABA content from MS4 to MS5, suggesting ABA content is closely associated with fruit ripening. Ethylene production decreased sharply from MS1 (287 nL/kg/h) to MS2 (14.5 nL/kg/h) and maintained relatively low output through the MS3, MS4, and MS5 stages (Fig. 1g). Our results indicated that mango fruit ripening mainly occurred from MS4 to MS5, including changes in TSS, firmness and color. Particularly, ABA content was closely associated with ripening through fruit development and ripening.

### Changes in metabolites with fruit development and ripening

**Figure 2:**
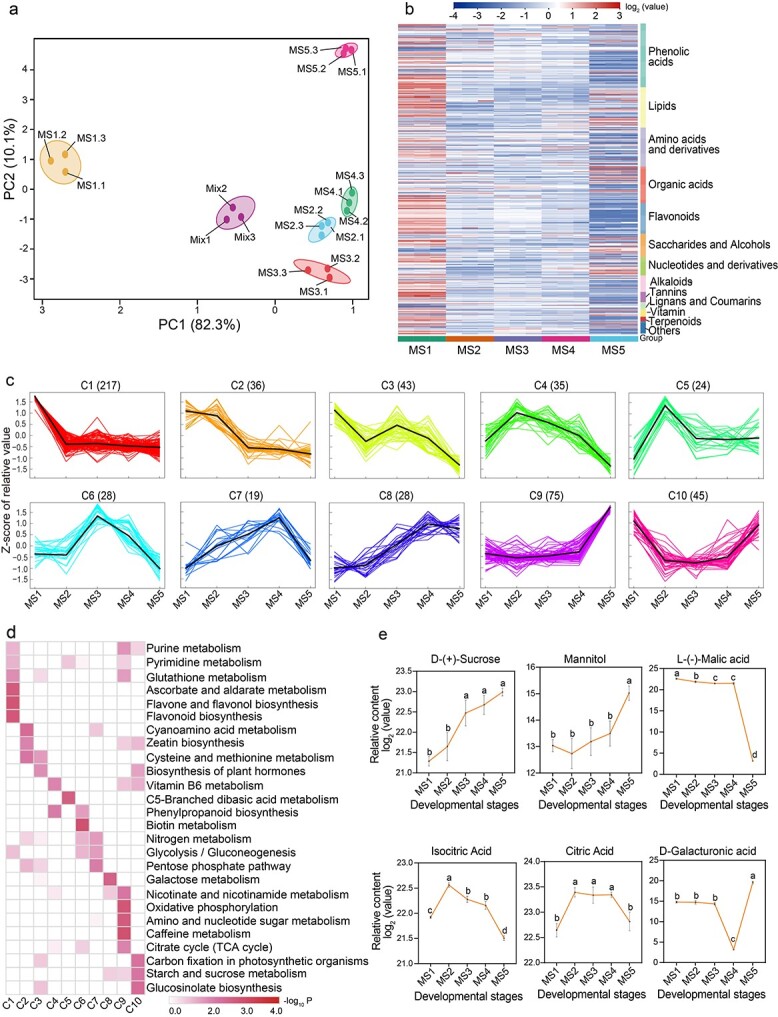
Metabolome analysis of five developmental stages in ‘Keitt’ fruit. **a** PCA of metabolome data. **b** Heat map of 617 metabolites from five developmental stages. **c**  *k*-Means clustering analysis on 550 DAMs. The *x* axis represents five key development stages (MS1–MS5) and the *y* axis depicts the *Z*-score of relative metabolite content. The numbers in parentheses are numbers of DAMs contained in the corresponding clusters (C1–C10). **d** KEGG enrichment analysis for DAMs among the 10 clusters. **e** Dynamic changes in key metabolites related to sugar metabolism and starch degradation pathway. Different letters stand for significant differences in different stages calculated through one-way ANOVA (*P* < .05). Error bars stand for the standard deviation of three replicates. Complete data can be found in Supplementary Data Tables S1–S3.

To investigate metabolite dynamics with fruit development and ripening, we employed a metabolomic identification at five stages (from MS1 to MS5). Principal component analysis (PCA) revealed that obvious differences in metabolite compounds accumulated at different stages (Fig. 2a). Specifically, metabolite compounds identified in the early stage (MS1) were distinct from those observed in other stages, and compounds of metabolites identified from MS2, MS3, and MS4 were similar. Metabolite compounds identified in the late stage (MS5) were distinct from those found in other stages. Further, hierarchical cluster analysis showed differences in metabolite compounds across the five stages (Supplementary Data Fig. S2a), exhibiting further distinctions in metabolite compound types between the early stage (MS1) and the middle stages (MS2, MS3, and MS4), as well as between the middle stage and the ripening stage, consistent with PCA analysis. Data reliability analysis revealed that metabolomic data were highly replicable [Pearson correlation (*r*_P_) ≥ .9] and qualified for further analyses (Supplementary Data Fig. S2b). In total, 617 metabolites were detected in at least one sample, including 126 phenolic acids, 80 lipids, 77 amino acids and derivatives, 72 organic acids, 61 flavonoids, 46 saccharides and alcohols, 36 nucleotides and derivatives, 33 alkaloids, 20 tannins, 15 lignans and coumarins, 14 vitamins, 9 terpenoids, 4 xanthones, and 24 other compounds (Supplementary Data Table S1). There were 609, 606, 605, 601, and 510 metabolites detected from the five stages (MS1–MS5), respectively. In particular, we noticed that metabolites were most abundant specifically in the early stages (MS1–MS2) and late-ripening stages (MS4 and MS5) (Supplementary Data Figs S2c and S3a), respectively. Further analysis found that MS1 compounds mainly comprised phenolic acids, flavonoids, alkaloids, and tannins (Fig. 2b), reflecting the biochemical accumulation in mango fruit in the early stage. MS5 mainly featured saccharides and alcohols, organic acids, and lipids, which were related to the formation of fruit flavor.

To investigate the accumulation dynamics of identified metabolites with fruit development and ripening, orthogonal partial least-squares discriminant analysis (OPLS-DA) was performed, resulting in 550 differentially accumulated metabolites (DAMs) throughout fruit development and ripening (Supplementary Data Table S2). Further, *k*-means clustering analysis exhibited 10 distinct clusters (C1–C10) corresponding to five different developmental stages: MS1 (C1–C3), MS2 (C4 and C5), MS3 (C6), MS4 (C7), and MS5 (C8–C10) (Fig. 2c; Supplementary Data Tables S2 and S3), suggesting that accumulation patterns of identified metabolites were diverse throughout fruit development and ripening. Specifically, C8, C9, and C10 reflected the significant increase in metabolites from MS4 to MS5. While inspecting the functions of clustered metabolites, we found that the clustered metabolites were involved in diverse metabolism and biosynthesis pathways (Fig. 2d). These analyses revealed that the metabolism pathways of purine, pyrimidine, glutathione, cyanoamino acid, cysteine, and methionine as well as the biosynthesis of flavones, flavonol, flavonoid, zeatin, and related hormones weakened from MS1 to MS2, and biosynthesis or metabolism of nicotinate, caffeine, sucrose, and galactose glucosinolate increased from MS4 to MS5. Increasing fruit sweetness along with decreasing acidity is a key indicator during fruit ripening. We were especially concerned with changes in factors that influence mango fruit sweetness and acidity. As shown in Fig. 2e, the contents of sucrose and mannitol exhibited an upward trend, while the levels of malic acid, isocitric acid, and citric acid descended as mango fruit ripened. Furthermore, we found that the level of galacturonic acid exhibited a dramatic increase from MS4 to MS5, which may be closely related to cell wall degradation (Fig. 2e). These metabolic and biosynthesis pathways determine changes in biochemical compounds in mango fruit development and ripening.

### Transcriptomic changes with fruit development and ripening

**Figure 3:**
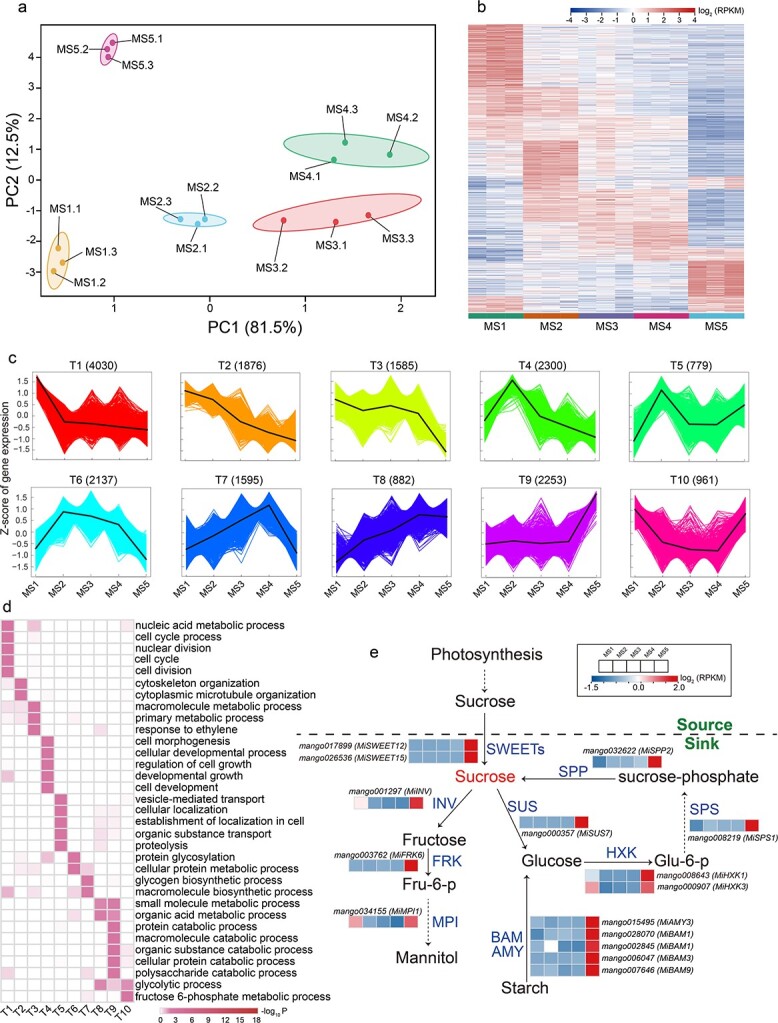
Global expression profile of five developmental stages in ‘Keitt’ fruit. **a** PCA of the transcriptome data. **b** Heat map of 21 349 genes from five developmental stages. **c**  *k*-Means cluster analysis on DEGs. The *x* axis represents five key development stages (MS1–MS5) and the *y* axis depicts the *Z*-score of standardized RPKM per gene. Numbers in parentheses are numbers of DEGs contained in the corresponding cluster. **d** GO enrichment analysis of 18 398 DEGs among the 10 clusters. **e** Expression pattern of structure genes involved in sucrose and starch metabolism during ‘Keitt’ fruit development and ripening. SPP, sucrose-phosphatase; SPS, sucrose-phosphate synthase; SWEET, bidirectional sugar transporter; SUS, sucrose synthase; INV, invertase; FRK, fructokinase; MPI, mannose-6-phosphate isomerase; HXK, hexokinase; BAM, β-amylase; DPE, 4-α-glucanotransferase; AMY, α-amylase. Complete data can be found in Supplementary Data Tables S5 and S7.

To uncover the potential molecular basis of fruit development and ripening, we conducted global transcriptomic analyses based on the RNA-seq technique. High-quality clean data were obtained for each tested stage of fruit development and ripening, with >94% clean reads uniquely mapped into the mango reference genome (Supplementary Data Table S4). We detected in total 21 349 genes expressed in at least one sample [with RPKM (reads per kilobase per million) >2]. PCA analysis showed that expressional differentiations of global genes were displayed across five stages (Fig. 3a). Specifically, the expressional differentiations of global genes were distinct among the five stages (Fig. 3b). Hierarchical cluster analysis again exhibited expressional differentiations of global genes from MS1 to MS5 (Supplementary Data Fig. S2d). Pearson correlation coefficient analysis showed that the transcriptomic data were highly replicable (*r*_P_ ≥ .85) across samples (Supplementary Data Fig. S2e). In particular, expressional differentiations of global genes were more striking from MS1 to MS2 and from MS4 to MS5 (Supplementary Data Figs S2f  and S3b).

In total, 18 398 differentially expressed genes (DEGs) were identified through five stages (Supplementary Data Table S5). The *k*-means clustering analysis exhibited 10 distinct clusters, named T1–T10 (Fig. 3c; Supplementary Data Table S5), highly similar to the clusters from metabolic analysis. While inspecting the potential roles of DEGs, the high-expression genes in MS1 (shown by T1, T2, and T3) and MS2 (shown by T4 and T5) were mainly involved in cell division and growth, such as in nuclear division, cell cycle and cytoskeleton, and cytoplasm organization, which may be related to the metabolic and biosynthesis processes involving purine, pyrimidine, flavonoid, phenylpropanoid, lignin and hormones enriched in the C1–C5 cluster (Fig. 2d; Supplementary Data Table S2); high-expression genes in MS3 (shown by T6) and MS4 (shown by T7) were mainly involved in protein glycosylation and glycogen and macromolecule biosynthesis processes (Fig. 3d; Supplementary Data Table S5), consistent with results from metabolome analysis, in which protein and sugar metabolisms occurred mainly at these two stages (Fig. 2d; Supplementary Data Table S2). The highest-expression genes from MS4 to MS5 were mainly involved in the biosynthesis and metabolism of glycogen and macromolecules (including sugars and proteins) such as small molecules and organic metabolic processes, organic acid catabolic processes, and polysaccharide catabolic processes, which are closely related to the fruit ripening process in mango (as shown by T8, T9, and T10) (Fig. 3d; Supplementary Data Table S5). This is in solid agreement with the increase in sugars (e.g. sucrose), decrease in organic acids (e.g. malic acid), and increase in d-galacturonic acid as the hydrolysate of the cell wall (Fig. 2e; Supplementary Data Table S2). Compared with metabolomic and transcriptomic data, our results revealed that the expressional differentiations of identified genes are highly associated with changes in metabolic accumulation with fruit development and ripening.

As shown in Figs 1d and 2e, TSSs (such as sucrose), a key indicator of mango fruit quality and taste, increased significantly during mango ripening, which mainly originates from the transport of photosynthates and the hydrolysis of starch. Our investigation also focused on the detection of potential key genes involved in these pathways. We detected 15 critical genes, including two bidirectional sugar transporter genes (*MiSWEET12* and *MiSWEET15*), one invertase gene (*MiINV*), one sucrose synthase gene (*MiSUS7*), one fructokinase gene (*MiFRK6*), one mannose-6-phosphate isomerase gene (*MiMPI1*), two hexokinase genes (*MiHXK1* and *MiHXK3*), one sucrose-phosphate synthase gene (*MiSPS*), one sucrose-phosphatase gene (*MiSPP2*), and five amylase genes (one *MiAMY3*, two *MiBAM1s*, one *MIBAM3* and one *MiBAM9*) related to sucrose metabolism and starch hydrolysis, which were specifically upregulated from MS4 to MS5 stage (Fig. 3e; Supplementary Data Table S5). In particular, the key sugar transporter genes *MiSWEET12* and *MiSWEET15*, responsible for sugar transportation and allocation within tissues as well as greatly contributing to fruit sweetness, were highly upregulated (up to 1910- and 15-fold, respectively) from MS4 to MS5. These genes, specifically expressed in the fruit ripening stage, could control mango sweetness formation.

Fruit softening, a critical indicator for fruit ripening, is tightly associated with the loosening and reduction of the fruit cell wall in cell–cell adhesion caused by a weakening of the pectin-rich middle lamella [41–43]. To identify the potential genes involved in controlling fruit softening, we identified 20 genes associated with cell wall loosening and primary cell wall degradation pathways that were specifically upregulated from MS4 to MS5, including five expansin genes (*MiEXPA1*, *MiEXPA2*, *MiEXPA6*, *MiEXPA8*, and *MiEXPB15*), five polygalacturonase genes (three *MiPGs* and two *MiQRT3s*), three pectate lyase genes (*MiPLY4*, *MiPLY5*, and *MiPLY8*), three pectinesterase genes (*MiPME1*, *MiPME53*, and *MiPPME1*), two rhamnogalacturonate lyase genes (two *MiRGLBs*), and two xyloglucan endotransglucosylase/hydrolase genes (*MiXTH8* and *MiXTH31*) (Supplementary Data Fig. S4a and Supplementary Data Table S5). Notably, the three pectate lyase genes responsible for pectin hydrolysis were highly and specifically upregulated from MS4 to MS5, resulting in a significant increase in the production of galacturonic acid (Fig. 2e; Supplementary Data Table S2).

In addition, the carotenoid content of pulp was often considered an important indicator of nutrients and pulp coloration in mango pulp [44–46]. Carotenoids were not detected in our samples owing to methodological limitations [47], but previous studies have revealed that the content of total carotenoids significantly increased during mango fruit ripening [34, 48]. While investigating potential DEGs involved in carotenoid biosynthesis, we revealed that 11 DEGs were highly and specifically expressed during fruit ripening (from MS4 to MS5; Supplementary Data Fig. S4b and Supplementary Data Table S5), including the geranylgeranyl pyrophosphate synthase gene (*MiGGPS*), the phytoene synthase gene (*MiPSY*), the phytoene desaturase gene (*MiPDS*), the zeta-carotene desaturase gene (*MiZDS*), two carotenoid isomerase genes (*MiCRTISO*), the lycopene beta-cyclase gene (*MiLCYB*), three beta-carotene hydroxylase genes (one *MiBCH1* and two *MiBCH2s*), and the zeaxanthin epoxidase gene (*MiZEP*). In particular, the expression of *MiPSY*, a key rate-limiting enzyme gene in carotenoid biosynthesis, was strikingly increased up to 8.3-fold from MS4 to MS5, indicating that these screened genes could be critical in the regulation of carotenoid content in mango fruit ripening.

### Abscisic acid plays a pivotal role in regulating mango fruit ripening

**Figure 4:**
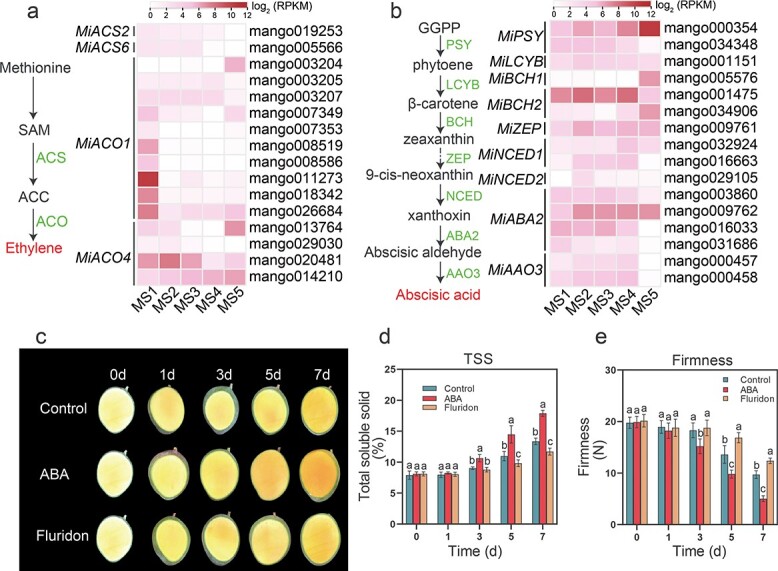
Effect of ethylene and ABA on ‘Keitt’ fruit ripening. **a** Ethylene biosynthesis pathway and the associated gene expression dynamics. Complete data can be found in Supplementary Data Table S6. **b** ABA biosynthesis pathway and expression trends of related genes. Complete data can be found in Supplementary Data Table S6. **c** Effect of external ABA and its inhibitor (fluridone) on ‘Keitt’ fruit ripening. Fruits were harvested at 165 DAB and treated with ABA and its inhibitor. Fruits were dissected from 0 to 7 days after treatment (0d–7d). **d** Dynamic changes in TSS under external ABA and its inhibitor application. **e** Impact of external ABA and its inhibitor application on fruit firmness. Different letters stand for significant differences in different stages calculated through one-way ANOVA (*P* < .05). Error bars stand for the standard deviation of three replicates.

According to the general hypothesis that climacteric fruit ripening is triggered and controlled by ethylene, the ripening process of mango fruit should also be triggered and regulated by ethylene. However, as shown in Fig. 1f and g, ethylene production mainly occurred at the early stage of fruit development, whereas ABA accumulation mainly occurred at the ripening stages, highly associated with the fruit ripening process. To dissect the potential regulator (ethylene or ABA) triggering mango fruit ripening, we investigated the profiles of DEGs involved in ethylene or ABA biosynthesis. The results indicated that a series of genes involved in ethylene biosynthesis were substantially downregulated with fruit ripening, consistent with the significant reduction of ethylene from MS1 to MS5 stages (Fig. 4a; Supplementary Data Table S6). Specifically, the key enzyme genes (*MiACS2* and *MiACS6*) encoding ACC synthases that control ethylene biosynthesis were not expressed with fruit ripening from MS4 to MS5. By contrast, many genes involved in ABA biosynthesis were obviously upregulated with fruit development and ripening, such as *MiPSY*, *MiBCH1*, and *MiNCED1* (Fig. 4b; Supplementary Data Table S6).

Moreover, we used exogenous ABA and its inhibitor (fluridone) to treat unmatured mango fruits *in vitro* at MS3 (165 DAB). As shown in Fig. 4c, ABA treatment speeded up the ripening process, whereas the fluridone treatment inhibited the ripening process. Correspondingly, the speed-up or delay of the fruit ripening process was physiologically reflected in the increase in total soluble solids and the reduction of firmness 3 days after ABA treatment, contrasting the three treatments among ABA, fluridone and control (Fig. 4d and 4e). To further investigate whether exogenous ethylene is able to affect the ripening process of ‘Keitt’ mango, we performed an exogenous treatment using ethylene and its inhibitor 1-methylcyclopropene (1-MCP). We found that ethylene could also accelerate the ripening process of ‘Keitt’ mango (Supplementary Data Fig. S5), though its modulation of ripening was slower compared with exogenous ABA treatment (Fig. 4c–e). Considering the changes in endogenous ABA production, the expression profiles of genes involved in the ABA biosynthesis pathway, and the physiological experiments, these results all clearly show that ABA played a critical role in triggering and regulating the mango fruit ripening process.

### Identification of potential genes related to abscisic acid-mediated fruit ripening

**Figure 5:**
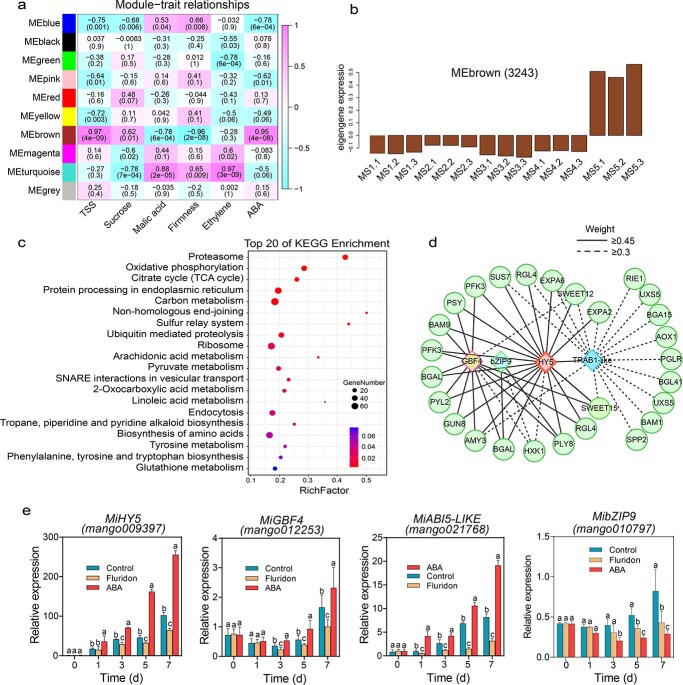
‘Keitt’ fruit ripening-related regulatory network and key genes. **a** Correlations between module and trait. The number in each cell represents the correlation coefficient and the *p* value. **b** Expression pattern of genes in MEbrown. **c** KEGG analysis of 3243 genes in the MEbrown module. **d** Co-expression network analysis of four bZIP-type TFs (*MiGBF4*, *Mi*b*ZIP9*, *MiHY5*, and *MiABI5-like*) and ripening-associated genes in the MEbrown module. Solid and dashed lines indicate the weighted Pearson correlation coefficient values for each gene pair. **e** Expression levels of *MiGBF4*, *MibZIP9*, *MiHY5*, and *MiABI5-like* after application of ABA and its inhibitor by RT–qPCR analysis. Different letters stand for significant differences in different stages calculated through one-way ANOVA (*P* < .05). Error bars stand for the standard deviation of five replicates. Complete data can be found in Supplementary Data Table S7. Primers are listed in Supplementary Data Table S10.

To uncover how ABA regulates the process of mango fruit ripening, we employed weighted gene coexpression network analysis (WGCNA) to build a co-expression network and identify the hub genes regulating mango fruit ripening. Among all the identified 21 349 expressed genes in developing and ripening fruit, we identified 10 distinct gene expression modules. Interestingly, the MEbrown gene set was significantly correlated with ABA accumulation and changes in fruit ripening-related traits included the contents of TSS, sucrose, and malic acid and firmness (Fig. 5a; Supplementary Data Table S7). The 3243 genes clustered in the MEbrown module showed relatively higher expression at the mango fruit ripening stage (MS5) (Fig. 5b; Supplementary Data Table S7). In the MEbrown module 3243 genes were enriched in diverse pathways such as proteasome, oxidative phosphorylation, protein processing in endoplasmic reticulum, TCA cycle, ubiquitin-mediated proteolysis, endocytosis, and many metabolic processes, such as linoleic acid, tyrosine, glutathione, pyruvate, 2-oxocarboxylic acid, nicotinate, and nicotinamide (Fig. 5c; Supplementary Data Table S7).

In total, we identified 70 TFs from the MEbrown module (Supplementary Data Table S8) that might regulate mango fruit ripening. Notably, we detected four hub ABA-responsive bZIP TF genes including *ELONGATED HYPOCOTYL 5* (*MiHY5*), *ABSCISIC ACID-INSENSITIVE 5-LIKE* (*MiABI5-like*), *G-BOX BINDING FACTOR 4* (*MiGBF4*), and *BASIC LEUCINE ZIPPER 9* (*MibZIP9*). The four bZIP TFs were highly expressed, with the highest expression of *MiHY5* occurring in the ripening stage (MS5) (Supplementary Data Fig. S6, Supplementary Data Table S8). The potential regulatory network mediated by these four bZIP TFs was constructed and is visualized in the Fig. 5d. We found that there was high connectivity between these TFs and many fruit ripening-related genes, such as *MiPSY*, involved in carotenoid biosynthesis, *MiPLY8*, involved in cell wall degradation, and *MiBAM9*, *MiHXK1*, *MiSWEET12*, and *MiSWEET15*, involved in the metabolic pathway of sugar and starch. In particular, *MiHY5* and *MiGBF4* were highly co-expressed with ripening-associated genes (Fig. 5d).

In order to further clarify whether the expressions of the four hub ABA-responsive TFs *MiHY5*, *MiGBF4*, *MiABI5-like*, and *MibZIP9* were induced by the ABA signal in mango fruit, we applied treatment with exogenous ABA and inspected the expression changes of *MiHY5*, *MiGBF4*, *MiABI5-like*, and *MibZIP9*. Results showed that *MiHY5*, *MiGBF4*, and *MiABI-like* were significantly induced by the ABA signal, and in particular *MiHY5* was upregulated 3.5-fold (Fig. 5e). However, the expression of *MibZIP9* was not induced via the exogenous ABA signal. While inspecting the expression changes of ripening-related genes, including *MiSWEET15*, *MiSWEET12*, *MiHXK1*, *MiPLY8*, *MiBAM9*, and *MiPSY*, which were most likely regulated by these TFs, we also found that they were significantly upregulated with exogenous ABA application, and their expressions were repressed with ABA inhibitor treatment (Supplementary Data Fig. S7). These results indicated that the ABA signal could directly induce the expressions of responsive factors (MiHY5, MiGBF4, and MiABI5-like), leading to the upregulation of ripening-related genes in mango fruit. Considering that the hub TF *MiHY5* is specifically and highly expressed in the fruit-ripening stage and substantially induced by the ABA signal, we subsequently verified the function of *MiHY5* in regulating the expressions of ripening-related genes.

### Confirmation of target genes directly regulated by abscisic acid-responsive MiHY5

**Figure 6:**
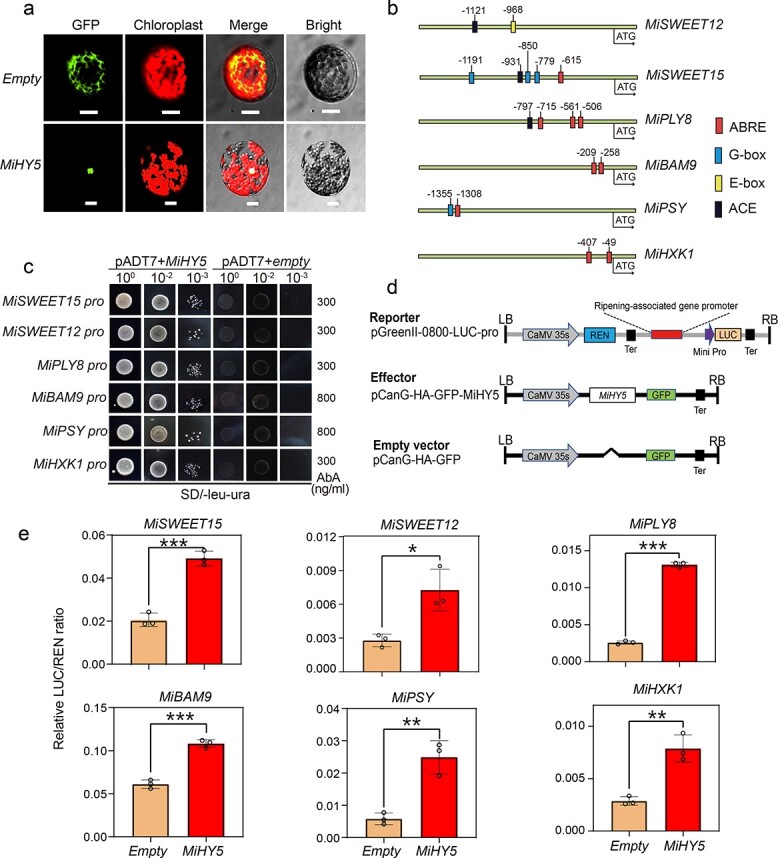
MiHY5 directly activates the expression of ‘Keitt’ fruit ripening-associated genes. **a** Subcellular localization of MiHY5. The empty vector pCanG-HA-GFP was used as a negative control. MiHY5 was found to be targeted to the cell nucleus. Scale bar indicates 20 μm. Images were taken in GFP channels (left) and bright field (right). **b** Schematic diagram of *cis*-elements in promoter region of six ripening-associated genes. ABRE, ABA response elements; G-box, *CACGTG*; E-box, *CAATTG*; ACE, *ACGT-containing element*. Complete data can be found in Supplementary Data Table S9. **c** A yeast one-hybrid assay showed that MiHY5 could bind to the promoters of ripening-associated genes. The empty vector pGADT7 was the negative control. SD medium was Ura and Leu double-deficient medium. Numbers on the right indicate the concentration of aureobasidin A. **d** Schematic diagram of vector construct. The reporter vector was pGreenII-0800-LUC-pro; the effector vector was pCanG-HA-GFP-MiHY5; the empty vector was pCanG-HA-GFP. **e** Activation by MiHY5 of six fruit-ripening associated genes by dual-luciferase assays in tobacco leaf protoplasts. Empty pCanG-HA-GFP vector was used as a negative control. Error bars stand for the standard deviation of three replicates. Asterisks represent significant differences calculated through one-way ANOVA (^*^P < .05, ^**^P < .01, ^***^P < .001). Primers are listed in Supplementary Data Table S10.

**Figure 7:**
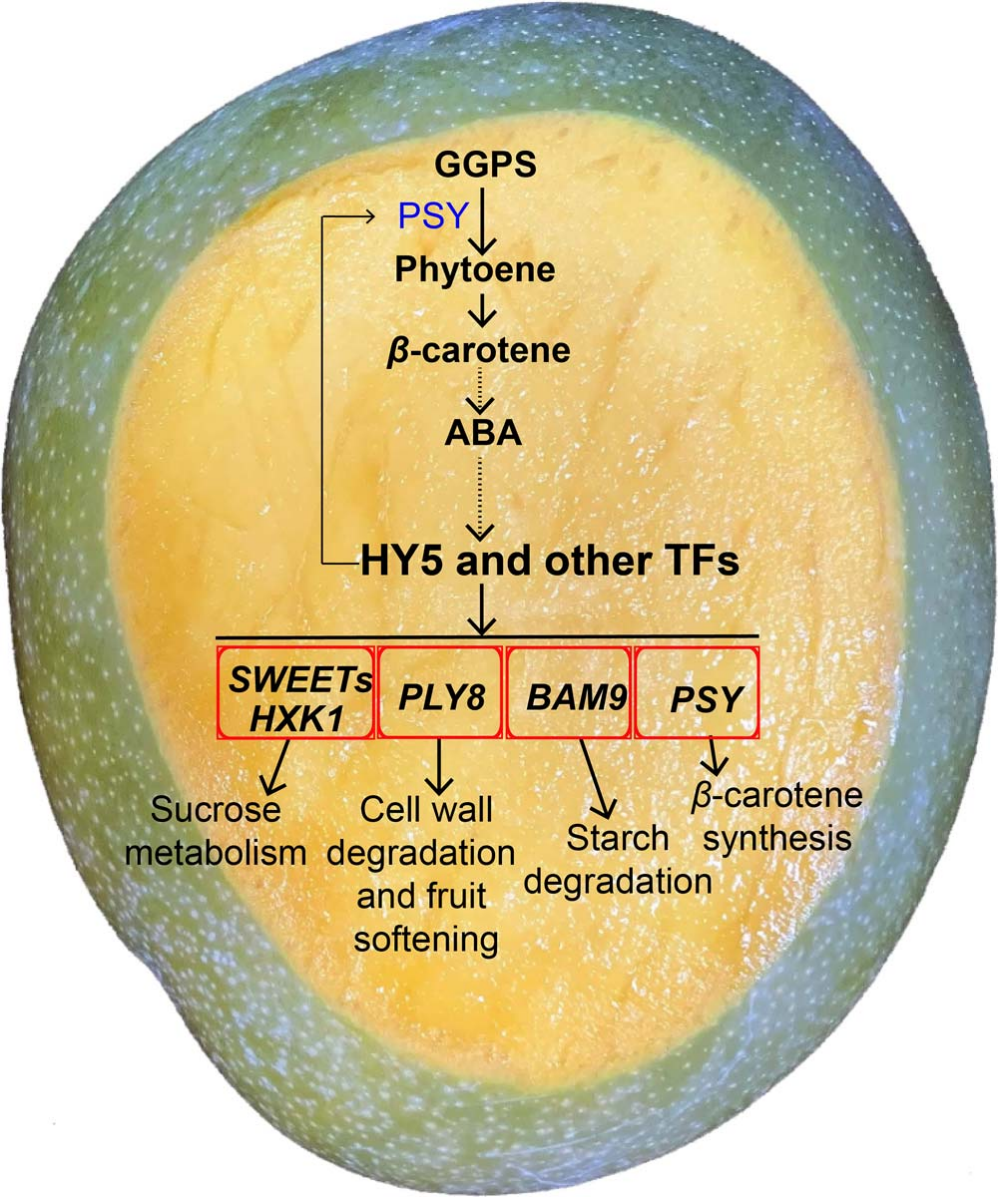
Model proposed for ABA-regulated ‘Keitt’ fruit ripening. With ‘Keitt’ fruit development, the content of endogenous ABA gradually increases and reaches a peak in the ripening stage. Endogenous ABA induces the expression of MiHY5 and other TFs. Subsequently, MiHY5 directly binds to the promoter *cis*-regulatory elements of ripening-associated genes to activate their expression. These ripening-associated genes are mainly involved in the sucrose metabolism process, cell wall degradation, fruit softening process, β-carotenoid accumulation, and starch degradation.

The TF HY5, a typical ABA signal-responsive factor, binds multiple *cis*-elements, such as G-boxes (CACGTG), E-boxes (CAATTG), ACE-boxes (ACGT), GATA-boxes (GATGATA), and ABREs (T/G/C ACGTG T/G), to activate the expression of target genes [49, 50]. According to the co-expression network (Fig. 5d), *MiHY5* most likely is the key regulator in controlling the expression of ripening-associated genes during fruit ripening. As shown in Fig. 6a, MiHY5 was exclusively localized in the cell nucleus via transient expression of tobacco leaf protoplasts, confirming the functional property of TFs in the nucleus. To test whether MiHY5 directly regulates the expression of ripening-associated genes, we selected six key ripening-associated genes identified above, including *MiSWEET15*, *MiSWEET12*, *MiHXK1*, *MiPLY8*, *MiBAM9*, and *MiPSY* for further experiments. We first cloned the promoter sequences (within 1.5 kb) of the six ripening-associated genes and detected several ABA responsive *cis*-elements within the 1.5-kb promoter region (Fig. 6b; Supplementary Data Table S9), implying potential binding between ABA-responsive TFs and these ripening-associated genes. As shown in Fig. 6c, MiHY5 could bind the promoters of six ripening-associated genes and activate their expression. Further, we constructed promoter-activation dual-luciferase reporter assay vectors (the pCanG-HA-GFP and pGreenII-0800-LUC vectors) (Fig. 6d) and performed a promoter activation experiment in tobacco leaf protoplasts. Based on the expression ratio of firefly LUC to Renilla luciferase (LUC/REN), we found significant changes in LUC/REN ratios for each tested gene between controls (empty vector without *MiHY5*) and the *MiHY5* constructed vectors (Fig. 6e). These results clearly showed that MiHY5 could directly bind to the promoters of six ripening-associated genes, thus activating their expression in tobacco leaf protoplasts. In short, both yeast-one-hybrid and promoter activation experiments confirmed that the ABA-responsive TF *MiHY5*directly regulates the expressions of ripening-associated genes during fruit ripening. Thus, we speculate that a functional pathway of MiHY5 in regulating mango fruit ripening exists via directly activating ripening-associated genes such as *MiSWEET*s, *MiHXK1*, *MiPLY8*, *MiBAM9*, and *MiPSY* (Fig. 7).

## Discussion

Fruit supply is commonly seasonal in markets. The duration of fruit development and ripening directly determines fruit shelf life and supply. The creation of diverse varieties with different durations of fruit development and ripening is critical for the breeding of fruit trees via genetic improvement in agriculture and forestry. Mango is one of the most popular and economically significant tropical fruits worldwide, widely distributed across the world. Many mangos are preclimacteric at harvest with commercial postharvest management. Dissection of the physiological and molecular mechanism of fruit development and ripening is quite important to service breeding practice and postharvest management. It is commonly believed that climacteric fruit ripening is mainly triggered by elevated ethylene levels, while the ripening of non-climacteric fruits is usually activated by an increase in ABA production with fruit development [12]. Although mango is considered a climacteric fruit, and application of exogenous ethylene obviously stimulated and hastened the ripening process [7, 39, 51, 52], endogenous ethylene production often varied greatly in different mango cultivars [53] or in different years in the same cultivar [54]. In particular, several studies have demonstrated that the increase in endogenous ABA toward ripening and exogenous ABA physiologically stimulate and hasten the mango ripening process [39, 55, 56]. The physiological function of ABA had been explained as a stimulator to initiate or induce ethylene biosynthesis, indirectly functioning in modulating mango fruit ripening [39, 55]. Based on changes in respiration rate with fruit development and ripening, ‘Keitt’ was considered a climacteric fruit in previous studies [57–59]. This study monitored the physiological and molecular changes in ‘Keitt’ fruit throughout the whole life of the fruit, providing an integrated and comprehensive profile for understanding the physiological and molecular basis of mango development and ripening. In particular, our current study clearly suggests that ABA plays a critical and direct role in modulating mango fruit ripening, suggesting the need for reframing the physiological function of ABA in modulating mango fruit ripening.

Similar to previous reports [39, 40, 55, 56], we observed that elevated endogenous ABA was closely associated with the process of mango fruit ripening, implying its physiological function is related to the modulation of fruit ripening. Although climacteric ethylene production might occur during fruit ripening, we observed ethylene biosynthesis in the fruitlet stage of ‘Keitt’. This ethylene profile might be related to its participation in regulating fruit growth and metabolic biosynthesis. Transcriptomic data clearly indicated that genes associated with ABA biosynthesis were upregulated with ABA accumulation, and many DEGs involved in ABA biosynthesis were identified during mango ripening, whereas genes related to ethylene biosynthesis were mainly expressed in the early stage, and DEGs associated with ethylene biosynthesis were not detected at the transitional stage of mango ripening (MS3–MS4 and MS4–MS5). Thus, our results do not support the explanation that ABA functions in modulating fruit ripening by initiating or inducing ethylene biosynthesis.

Our metabolomic analyses isolated 617 metabolites from different developmental stages of ‘Keitt’ mango fruit. Combined with the 181 volatiles identified from different stages of ‘Tainong’ mango fruit [60], the metabolites identified in this study greatly enrich existing information on metabolites identified from developing mango fruits, and include most of the metabolites identified in other cultivars [61]. Many identified metabolites were stage-specific, especially the distinct metabolites identified from the ripening stages embodied the accumulation of sugar, organic acids and other taste components at this stage. These metabolites identified from the ripening stage reflect, to a great extent, the chemical basis of mango nutrient composition.

Highly correlated with metabolomic patterns at different development stages, transcriptomic investigation also revealed two distinct transitions, from MS1 to MS2 and from MS4 to MS5, suggesting two distinct physiological courses that occur from the fruitlet stage to the fast-enlarging stage as well as the middle to late stages of fruit ripening. Transcriptomic analyses, in total, identified 21 349 unique transcripts with 18 398 DEGs during transitions between different development stages. DEGs identified from the fast-enlarging stage were functionally enriched in diverse enzymes and regulators that might be critical in the regulation of fruit development. Many DEGs identified from the late stage of ripening were functionally involved in the formation of softening and biosynthesis of featured metabolites (referring to ripening maker metabolites, e.g., sucrose, $\beta$-carotene, galacturonic acid and so on). These DEGs functionally associated with the biosynthesis of important metabolites such as sugar and carotenoids could be candidate genes responsible for mango fruit quality and taste. Furthermore, a number of DEGs associated with ABA biosynthesis (e.g. *MiPSY*, *MiBCH1*, and *MiNCED1*) were upregulated, and correspondingly many DEGs in the ABA pathway were upregulated during fruit ripening. Furthermore, the co-expression analysis detected ABA-responsive regulators such as *MiHY5*, *MiGBF4*, *MiABI5-like*, and *MibZIP9*, and their connection network with ripening-related genes. In particular, both the exogenous ABA application and experimental confirmation clearly revealed that the ABA signal directly modulated mango fruit ripening. Studies have revealed that HY5 can modulate fruit ripening and coloration, contributing to fruit qualities in other plant species, such as tomato [62, 63], peach [64], litchi [65], and apple [66]. It is likely that TF HY5 is functionally conserved in regulating fruit ripening through an ABA-dependent pathway. In contrast, the low expression of key genes in ethylene biosynthesis and the inactivated ethylene pathway were identified during mango fruit ripening. In summary, our results provide sound evidence that ABA acts in directly modulating mango fruit ripening.

The physiological organization and molecular modulation of fruit ripening is complicated and often species-specific. Indeed, mango is a climacteric fruit featuring great variation of endogenous ethylene production among different cultivars [67]. ‘Keitt’ mango, with climacteric and late-ripening behavior, has a relatively lower respiration rate and lower ethylene production compared with other varieties (early or middle ripening varieties) [68, 69]. Our results here verified the low ethylene production during fruit ripening, but exogenous ethylene was able to accelerate the ripening process of ‘Keitt’ (Supplementary Data Fig. S5), though it was slower compared with the effect of ABA (Fig. 4). Since ABA takes part in directly modulating ‘Keitt’ mango fruit ripening with low ethylene production, how the low respiration rate is induced during fruit ripening remains unknown. Whether or how ethylene and ABA cross-talk or interact to modulate ‘Keitt’ mango fruit ripening is also uncertain. In particular, we noticed that ‘Keitt’ has a typical late-ripening behavior with a longer duration of fruit development and ripening than other early- or middle-ripening varieties. Whether the ripening behavior of mango fruit is related to the different modulations of ABA or ethylene deserves further investigation. Thus, whether the critical and direct role of ABA in modulating mango fruit ripening is common or cultivar-specific remains unclear.

## Materials and methods

### Plant materials

‘Keitt’ mango, a late-ripening and widely cultivated variety that undergoes a longer ripening stage than most early- or middle-ripening cultivars [68], was investigated in this study. Approximately 15-year-old mango trees grown in a commercial orchard (Yanbian County, Panzhihua City, Sichuan Province, 26°43′26″ N, 101°48′54″ E, altitude 1314.7 m) were used as materials. All fruit samples were collected from 30 trees in 2019. Fruits were collected every 15 days (one fruit per tree at a time) from bloom to fruit fully ripening. According to the fruit size and color, five development stages were marked 45, 105, 165, 195 and 240 (fully ripe) DAB, harvested, and named MS1, MS2, MS3, MS4, and MS5. At each sampling point, healthy fruits were selected for consistent appearance. The fresh fruits were immediately used for physiological property measurements (fruit weight, size, total soluble solids, total acid, firmness, ethylene production, and ABA content), and fruit pulp samples were harvested for further analysis.

### Physiological property measurement

Fruit weight, length, width, and thickness were measured using an electronic balance and a Vernier caliper (30 replicates per stage). TSS was measured using a refractometer (PAL-1, ATAGO). TA was measured referring to a formerly reported method [7]. Fruit pulp firmness was determined with a GY-4 texture analyzer (Yueqing Handpi Instruments Co., Ltd) with three biological replicates (five fruits per replicate). Ethylene release of each developmental stage was measured by placing individual fruits in a 2-L airtight container for 2 hours at 22°C. The obtained gas samples were analyzed on a gas chromatograph (Agilent 7890A). Ethylene production was calculated by comparison with the standard curve and normalized according to fruit weight. Endogenous ABA was quantified following a method published previously [70] with minor revision, and 5 ng of ^2^H_6_-ABA was used as internal standard. Three replicates were used for ethylene and ABA quantification in each group.

### Widely targeted metabolomics analysis

Extract preparation and metabolite profile characterization of mango fruit pulp were carried out by Wuhan Metware Biotechnology Co., Ltd (Wuhan, China) following the standard processes [71, 72]. Briefly, ~100 mg of fruit pulp sample was extracted with 70% aqueous methanol, and the extracted sample was analyzed with a UPLC–ESI–MS/MS system. Simultaneously, separated metabolites were subjected to ESI–MS/MS. Three replicates were used for all samples. Quantification and annotation of metabolites was performed through a scheduled multiple reaction monitoring method [73]. Metabolic profiles during mango development were visualized by TBtools [74].

### Transcriptome sequencing

Total RNA was extracted from pulp at five developmental stages of ‘Keitt’ (three replicates per stage). In brief, raw data were obtained using the Novaseq 6000 platform. The clean data were filtered from the raw data by Trimmomatic (version 0.36) and then aligned to the mango reference genome (cultivar ‘Hong Xiang Ya’) [75] using STAR v2.5.3a [76]. Gene expression was normalized into RPKM using RSEM (v.1.3.0) [77]. DEGs were screened by a fold-change ≥2 and *P*-value ≤.05 by applying DEseq2 [78]. *k*-Means cluster analysis was conducted using an R package [79]. All the raw reads have been submitted to NCBI under accession number PRJNA773197. The expression patterns of genes were generated by TBtools [74].

### Weighted gene co-expression network analysis

WGCNA (v1.66) was applied to construct unsigned co-expression networks based on the gene expression matrix (RPKM ≥2 at least in a sample) [80]. The parameters were as follows: the soft threshold power was 14; the minModuleSize was 30; the cutHeight was 0.25. The eigengene value was calculated for each module and used to test the association with each sample or traits. OmicShare tools (https://www.omicshare.com/tools) were used to perform GO (Gene Ontogeny) and KEGG (Kyoto Encyclopedia of Genes and Genomes) enrichment analysis. The co-expression network was visualized with Cytoscape (v3.8.1) [81].

### Exogenous abscisic acid and ethylene treatment assay

In total, 150 mature green mango fruits harvested at MS3 were randomized into five groups. Fruits were washed and sterilized with 1% sodium hypochlorite and air-dried. For exogenous ABA treatment, ABA and its inhibitor (fluridone) were prepared by dissolving them in ddH_2_O, and Tween-20 was used as adherent. Then, fruits of groups 1, 2, and 3 were respectively dipped in water (as a control, with the same concentration of Tween-20), ABA (1 mM/L), and fluridone (200 μM/L) for 10 minutes at 25°C. For ethylene treatment, the pretreatment of all fruits was the same as that of the above control group, and then fruits of groups 4 and 5 were respectively treated with ethylene (100 μL/L) and 1-MCP (1 μL/L) in airtight containers at 25°C. Twenty-four hours later, all samples of each group were stored in a container at 25°C, with relative humidity 85 ± 5%. The selected physiological parameters mentioned above were recorded for each group at 2-day intervals, with three biological replicates at each time point.

### RT–qPCR analysis of gene expression

RT–qPCR was performed in the Perfect Start™ Green RT-qPCR Super Mix system (TransGen), with five biological replicates. *MiARF* (mango002359), encoding an ADP-ribosylation factor-like protein, was used as the endogenous reference gene [82]. The primers used in this study are listed in Supplementary Data Table S10.

### Identification and functional characterization of *MiHY5* in mango

The full-length coding sequence (CDS) of *MiHY5* was isolated by PCR amplification using Phanta Max Super-Fidelity DNA Polymerase (Vazyme, Nanjing, China). The CDS was next confirmed via Sanger sequencing. The primers are listed in Supplementary Data Table S10. To investigate the subcellular localization of MiHY5, we performed transient expression in tobacco (*Nicotiana benthamiana*) leaf protoplast. Briefly, full-length CDS was cloned from mango fruit, digested with XbaI and ligated into the pGanG-HA-GFP vector using the CloneExpress^®^ II One Step Cloning Kit (C112; Vazyme). The constructed plasmid was transformed into *Agrobacterium tumefaciens* strain GV3101 (pSoup-p19). An *A. tumefaciens* strain containing plasmids of interest was transiently expressed in tobacco leaf epidermis via a leaf injection procedure [83]. At the same time, an *A. tumefaciens* strain with empty vector was used as the control. Following incubation at 22°C for 3 days, protoplasts were prepared from tobacco leaf epidermis [84] and observed under a fluorescence microscope (FV1000, Olympus, Japan).

### Yeast one-hybrid assays

Promoter fragments around the ABRE *cis*-elements of six fruit ripening-related genes were cloned via PCR using corresponding primers (Supplementary Data Table S10). Amplified products were recognized by Sanger sequencing and cloned into the SacI and HindIII sites of pAbAi vector as the baits, and the full-length CDS of *MiHY5* was fused in the NdeI and EcoRI sites of the pGADT7 vector to construct the prey. The recombinant vectors were then transformed into the yeast Y1H Gold strain via the homologous recombination method, and further selected for resistance concentrations using SD/−Leu−Ura with proper concentrations of aureobasidin A. The protein–DNA interaction was determined according to growth ability of the yeast cells.

### Dual-luciferase assays

The full-length CDS of *MiHY5* was cloned into the XbaI site of the pCanG-HA-GFP vector under the control of the CaMV *35S* promoter as the effector. The promoter fragments mentioned above were ligated into the SalI and KpnI sites of the binary vector pGreenII 0800-LUC as the double-reporter vector [85]. The pCanG-HA-GFP vector without *MiHY5* was used as negative control. Primers used in this assay are listed in Supplementary Data Table S10. The constructed effector and reporter vectors were co-transformed into tobacco leaf (*N. benthamiana*) mesophyll protoplasts using the polyethylene glycol (PEG) method as previously reported [84]. The transformed protoplasts were incubated at 23°C for 16 hours in darkness, and dual-luciferase assays were performed using the Dual-Luciferase^®^ Reporter Assay System (Vazyme, China). Luciferase activity was determined using the Infinite^®^ 200 PRO plate reader (Tecan Group, Switzerland). Finally, the LUC:REN ratio was calculated and normalized to the control vector as the final value. Dual-luciferase assays were repeated three times.

## Acknowledgements

This work was supported by the Department of Science and Technology of Yunnan, China (Grant No. 202003 AD150004). We are grateful to the Service Center for Experimental Biotechnology at the Kunming Institute of Botany, CAS, for supporting plant cultivation and instrument. We are also grateful to Dr Fei Li (Service Center for Experimental Biotechnology) for his assistance in ethylene production analysis.

## Author contributions

A.L., W.X., S. Wang, S. Wu, and J.X. conceived and designed the experiments; S. Wu, D.W., J.S., and Y.Z. performed the experiments; S. Wu, J.S., Q.T., and J.Y. performed field observations and sample collection; J.S. and T.Y. cultivated tobaccos; S. Wu, A.L., and W.X. analyzed the data and wrote the manuscript.

## Data availability

All the RNA-seq data are available and have been deposited in the National Center for Biotechnology Information Sequence Reads Archive (SRA) with accession number PRJNA773197.

## Conflict of interest

The authors declare that they have no conflict of interest.

## Supplementary data

Supplementary data is available at *Horticulture Research * online.

## Supplementary Material

Web_Material_uhac102
