# Supplementary figures and images for: Metabolomic and transcriptomic analyses reveal new insights into the role of abscisic acid in modulating mango fruit ripening
Source: Hortic Res. 2022 May 10;9:uhac102. doi: 10.1093/hr/uhac102 (PMC9250656; doi:10.1093/hr/uhac102)

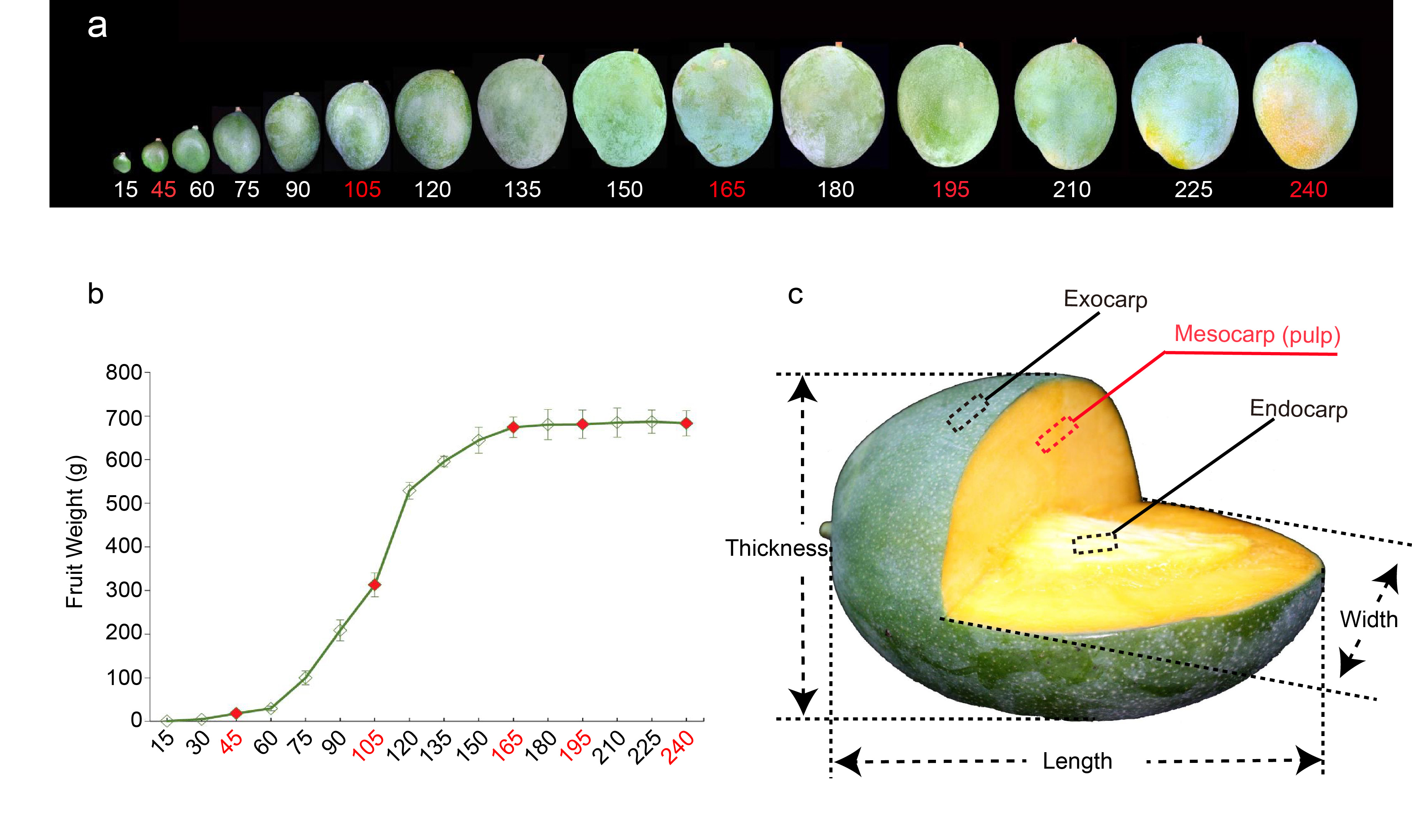

Supplement: Web_Material_uhac102 [file web_material_uhac102.zip › Figure S1.jpg]

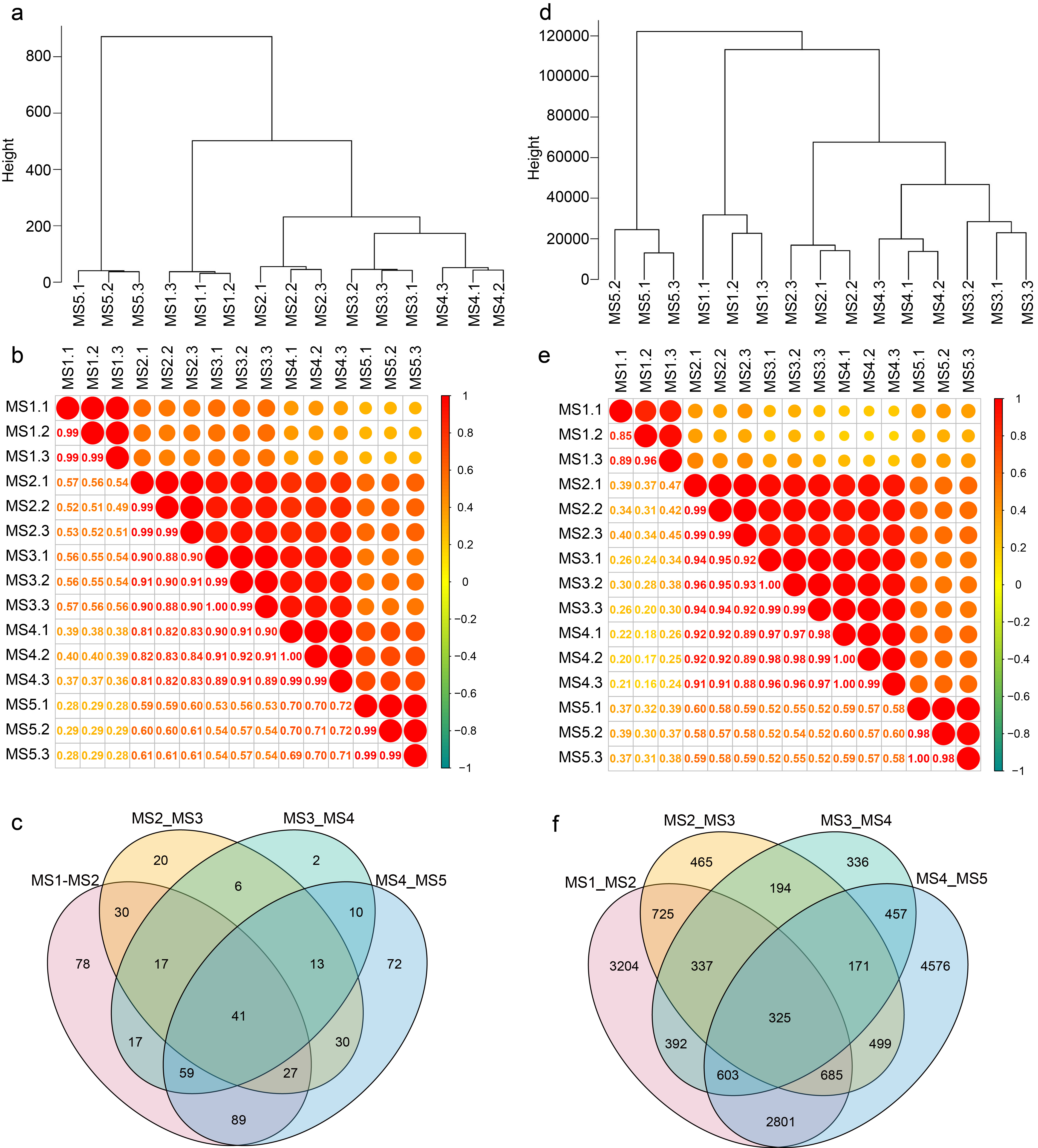

Supplement: Web_Material_uhac102 [file web_material_uhac102.zip › Figure S2.jpg]

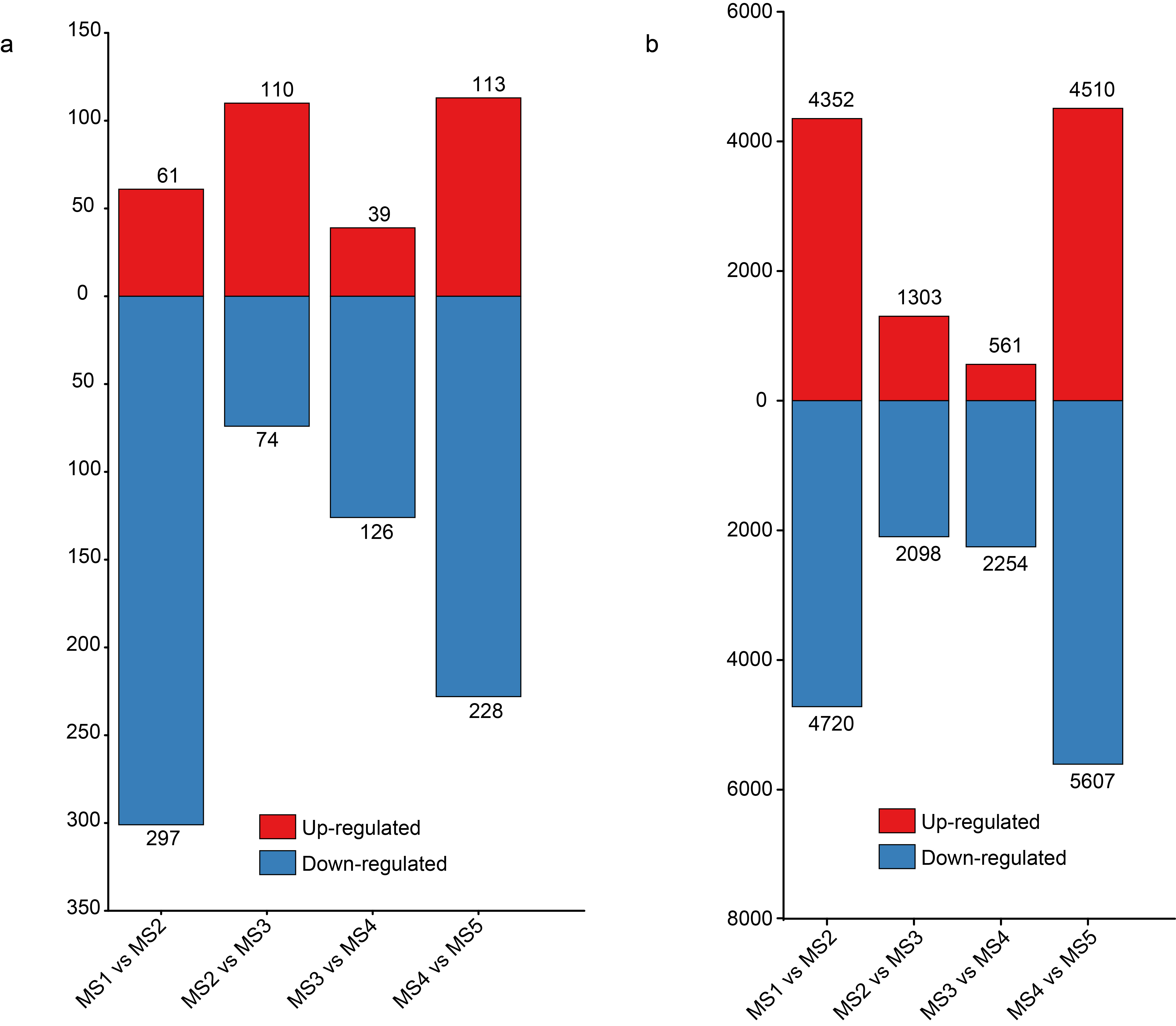

Supplement: Web_Material_uhac102 [file web_material_uhac102.zip › Figure S3.jpg]

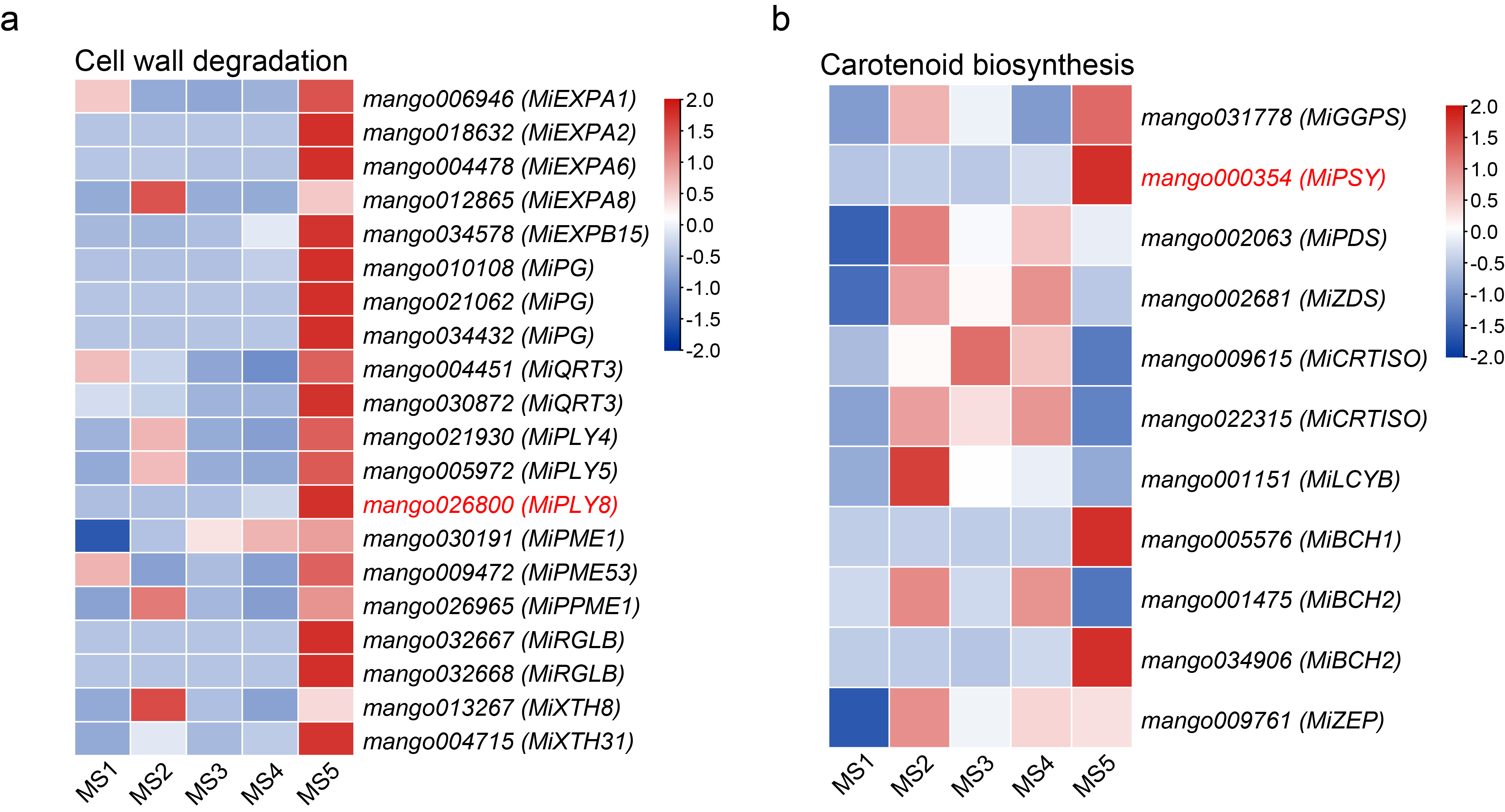

Supplement: Web_Material_uhac102 [file web_material_uhac102.zip › Figure S4.jpg]

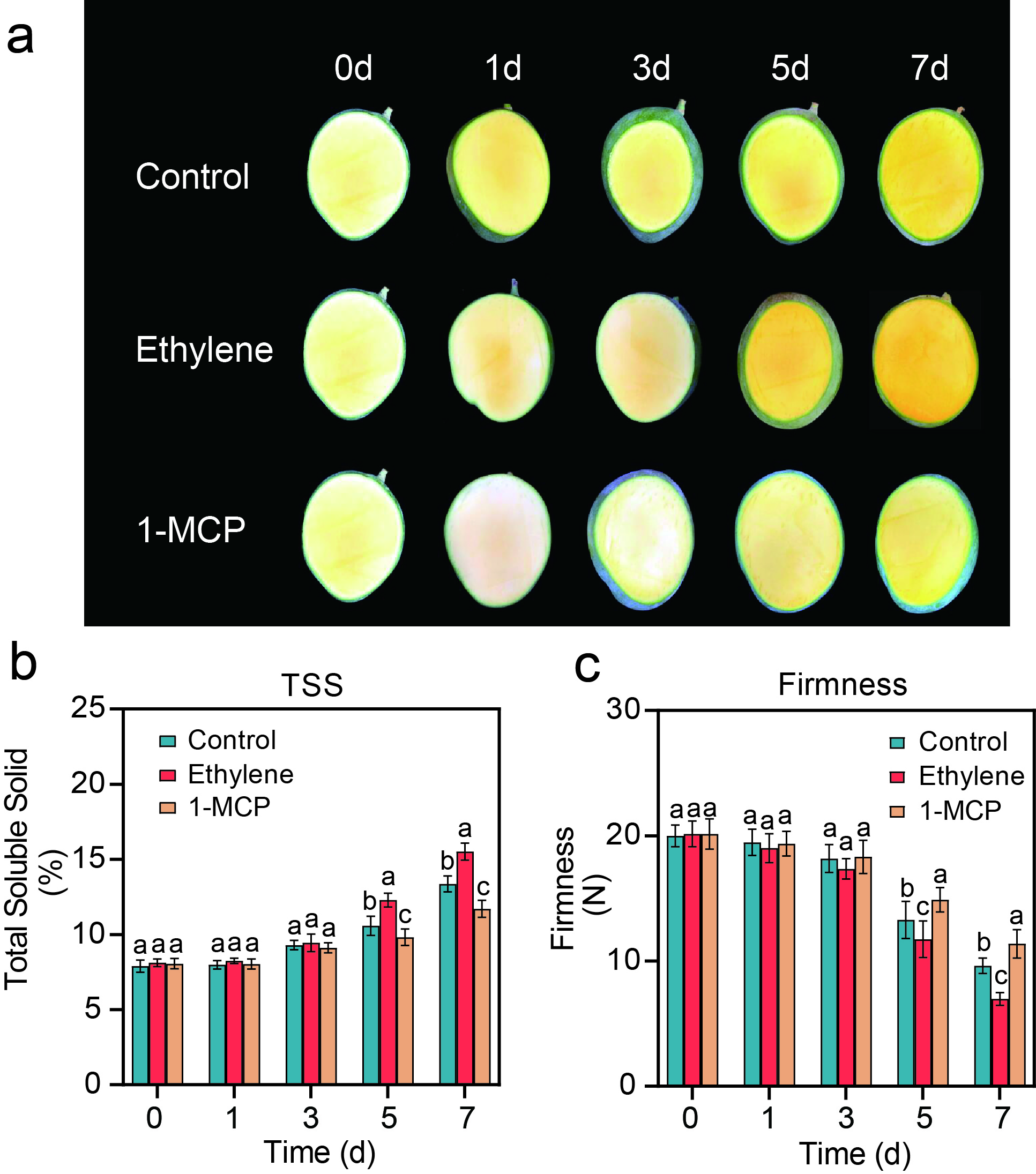

Supplement: Web_Material_uhac102 [file web_material_uhac102.zip › Figure S5.jpg]

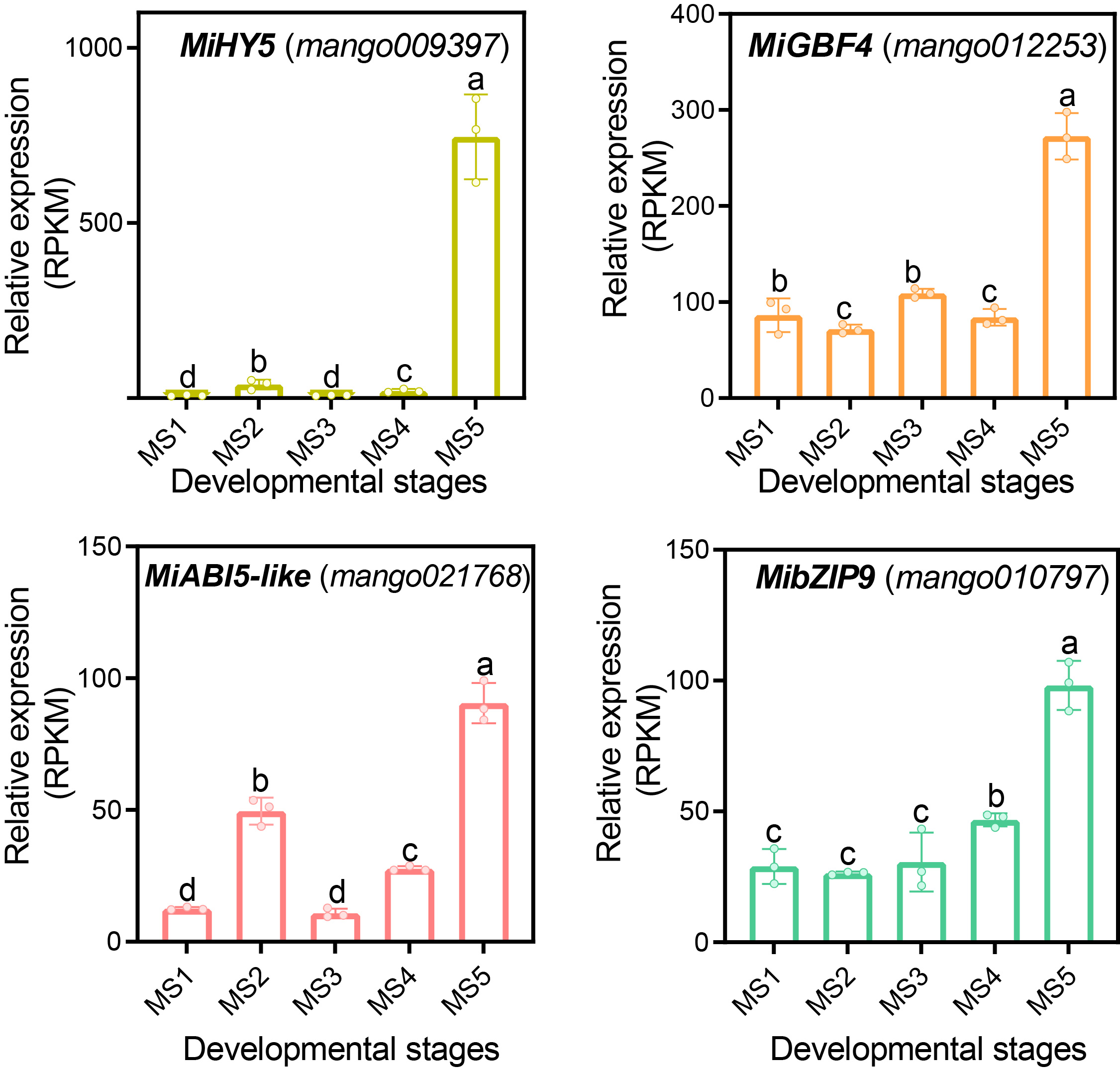

Supplement: Web_Material_uhac102 [file web_material_uhac102.zip › Figure S6.jpg]

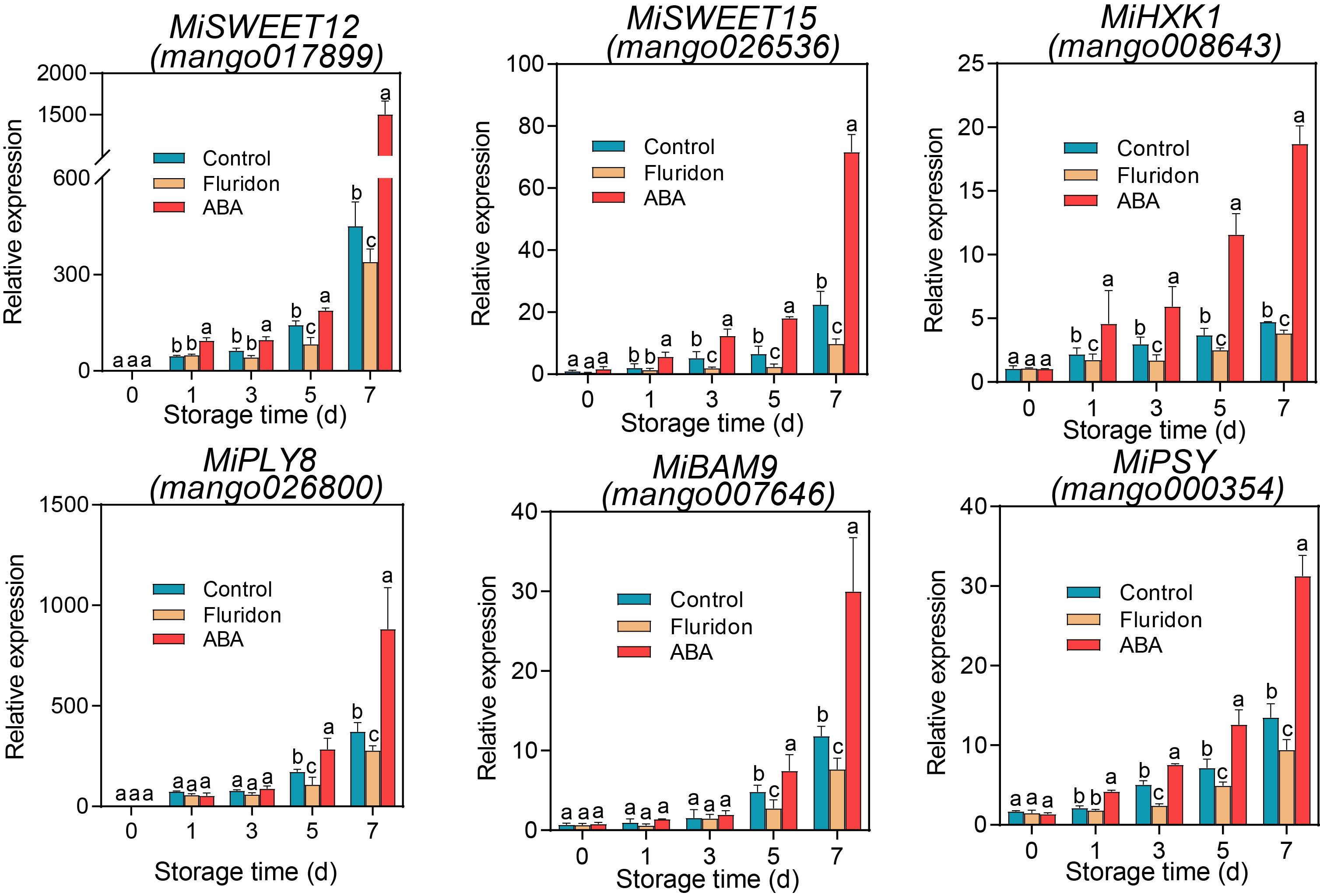

Supplement: Web_Material_uhac102 [file web_material_uhac102.zip › Figure S7.jpg]
